# Grafting of Hindered Phenol Groups onto Ethylene/α-Olefin Copolymer by Nitroxide Radical Coupling

**DOI:** 10.3390/polym9120670

**Published:** 2017-12-04

**Authors:** Serena Coiai, Francesca Cicogna, Chengcheng Yang, Veronika Tempesti, Sabrina Carola Carroccio, Giuliana Gorrasi, Raniero Mendichi, Nadka Tz. Dintcheva, Elisa Passaglia

**Affiliations:** 1 Istituto di Chimica dei Composti Organo Metallici (ICCOM), Consiglio Nazionale delle Ricerche, SS Pisa, Via G. Moruzzi 1, 56124 Pisa, Italy; serea.coiai@pi.iccom.cnr.it (S.C.); chengchengyoung@gmail.com (C.Y.); v.tempesti@outlook.it (V.T.); passaglia@pi.iccom.cnr.it (E.P.); 2 Istituto per i Polimeri, Compositi e Biomateriali (IPCB), Consiglio Nazionale delle Ricerche, SS Catania, Via P. Gaifami 18, 95126 Catania, Italy; sabrinacarola.carroccio@cnr.it; 3 Istituto per la Microelettronica e Microsistemi (IMM), Consiglio Nazionale delle Ricerche, SS Catania (Università), Via S. Sofia 64, 95123 Catania, Italy; 4 Dipartimento di Ingegneria Industriale, Università Degli Studi di Salerno, Via Giovanni Paolo II, 132, 84084 Fisciano (SA), Italy; ggorrasi@unisa.it; 5 Istituto per lo Studio delle Macromolecule (ISMAC), Consiglio Nazionale delle Ricerche, Via A. Corti 12, 20133 Milano, Italy; mendichi@ismac.cnr.it; 6 Dipartimento di Ingegneria Civile, Ambientale, Aerospaziale, dei Materiali, Università di Palermo, Viale delle Scienze, Ed. 6, 90128 Palermo, Italy; nadka.dintcheva@unipa.it

**Keywords:** antioxidant covalent immobilization, nitroxide radical coupling, hindered phenol moiety, HAS-NOR antioxidant

## Abstract

The covalent immobilization of hindered phenol groups, with potential antioxidant activity, onto an ethylene/α-olefin (EOC) copolymer was carried out by the nitroxide radical coupling (NRC) reaction performed in the melt with a peroxide and the 3,5-di-*tert*-butyl-4-hydroxybenzoyl-2,2,6,6-tetramethylpiperidine-1-oxyl radical (BHB-T). Functionalized EOC (EOC-*g*-(BHB-T)) was exposed to photo- and thermo-oxidation. By comparison with some model compounds bearing the (2,2,6,6-tetramethylpiperidin-1-yl)oxyl (TEMPO) moiety or the hindered phenol unit, it was observed that the grafted BHB-T could effectively help the stabilization of the polymer matrix both under photo- and thermo-oxidation. In addition, the immobilization of BHB-T can effectively increase the service life of the functionalized polymers when polymer films were put in contact with ethanol solution thus simulating a possible application of the modified polymer.

## 1. Introduction

The addition of antioxidants (AOs) to protect polymers from oxidation induced by light or temperature is a widely studied subject because oxidative processes can cause the severe loss of mechanical, thermal, and rheological properties of polymer matrices [1,2,3]. To limit or control the loss by evaporation or leaching of low molecular weight AOs added to the polymer matrix, different strategies have been applied like for example the use of AOs bearing long alkyl chains that are less volatile and more affine to the polymer matrix, or the immobilization of AOs onto solid supports or onto the polymer backbone. For example, hindered phenol groups were anchored onto silica nanoparticles [4,5], intercalated into layered double hydroxides [6,7,8] or montmorillonite clay [9] and used as stabilizers to prevent the oxidation of poly(propylene) (PP), poly(ethylene) (PE) [4,5,6,7,9] and also poly(lactic acid) (PLA) [8]. Alternatively, the literature reports examples of AO immobilization onto polymer matrices by formation of a covalent bond. The grafting of AOs was obtained by modification of a pre-formed polymer by a radical functionalization procedure [10,11,12,13] or by copolymerization between functionalized and un-functionalized monomers [14,15,16,17,18]. 

Acryloyl or methacryloyl monomers as well as bis-maleate and maleimide, all substituted with AO groups, were grafted to different polymer matrices [10,11,13]. For example maleate derivatives bearing hindered amine groups were covalently bonded to PP by radical grafting [19], whereas maleimides substituted with a hindered phenol group were grafted to PE and PP [13]. An alternative to post-polymerization modification is the polymerization or co-polymerization of *ad hoc* chosen monomers, like for example the polymerization of monomers bearing hindered phenol groups [17] or the copolymerization of ethylene with AO functionalized norbornene [14,15,16,17,18]. Although the aforementioned studies have demonstrated the possibility to immobilize AO units on polymers, they all suffer from a few drawbacks. For example, the post-polymerization modification is generally accompanied by side reactions that are not easy to control [20]. On the other hand, the polymerization or co-polymerization approach generally requires a careful choice of the polymerization pathways (radical or catalyzed route) that depends on the relative reactivity and polarity of the involved vinyl monomers as well as on the final composition and architecture of the desired material [21]. Probably for these reasons, most of the papers that describe the immobilization of AO groups onto a polymeric matrix are focused on the preparation of materials rather than on their real antioxidant features [10,11]. When this is discussed, generally a good antioxidant efficiency of the immobilized AO is reported that is, in some cases, even higher than that observed for corresponding free AOs. However, these results are generally ascribed to the higher concentration of immobilized AO, which cannot be lost during the preparation of the sample, rather than to its higher efficiency [4,5,12].

Among the post-polymerization methods, the nitroxide radical coupling (NRC) reaction is a very interesting tool to graft specific functionalities onto polymer chains providing an excellent control of macroradical formation versus grafting [22,23,24,25,26,27,28]. 

**Scheme 1:**
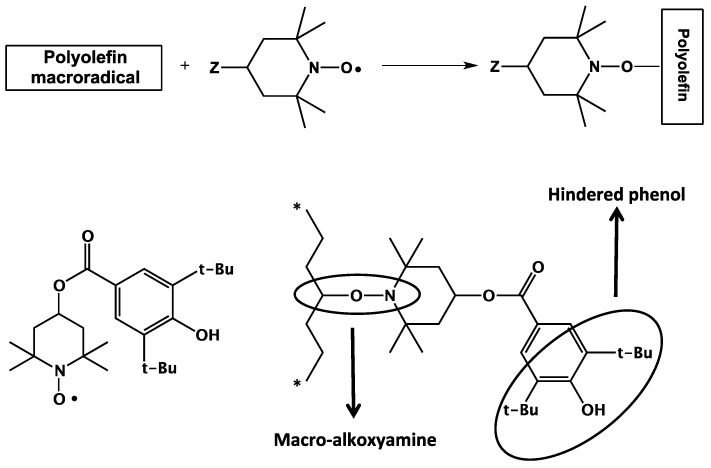
Schematic representation of nitroxide radical coupling (NRC) reaction, structure of 3,5-di-*tert*-butyl-4-hydroxybenzoyl-2,2,6,6-tetramethylpiperidine-1-oxyl radical (BHB-T) and possible double role of ethylene/α-olefin (EOC) functionalized with (BHB-T) ((EOC-*g*-(BHB-T)) in the antioxidant protection of the polymer matrix.

This reaction is initiated by peroxide decomposition and occurs in a single step with a very good control of macromolecular architecture. Moreover, as previously demonstrated, the NRC reaction is a suitable method to graft different functional nitroxides bearing esters, alcohol, and chromophores to PE and polyesters [22,23,24,25]. Thanks to all these positive features, this functionalization method was used here to immobilize onto a copolymer ethylene/α-olefin (EOC) the 3,5-di-*tert*-butyl-4-hydroxybenzoyl-2,2,6,6-tetramethylpiperidine-1-oxyl radical (BHB-T) bearing the 3,5-di-*tert*-butyl-4-hydroxybenzoyl antioxidant moiety and the (2,2,6,6-tetramethylpiperidin-1-yl)oxyl (TEMPO) group combined within the same molecule (Scheme 1). The p-hydroxybenzoate derivatives are described as chain breaking AOs like the 2,6-di-*tert*-butyl-4-methylphenol. However, due to the presence of the ester group (an electron-withdrawing substituent) in the para position with respect to OH [29], they are reported to be able to react with highly reactive species such as hydroxyl (HO^.^) and alkoxy radicals (RO^.^) produced from the decomposition of hydroperoxides (POOH) [30]. This feature of the phenolic hydrogen limits also its interaction with TEMPO radicals that instead are reported to react with other hindered phenol species giving phenoxyl radicals, with reduced AO activity, and hydroxylamine TEMPO [30,31]. The free nitroxide functionality of the BHB-T molecule, in agreement with the NRC reaction, was here exploited to graft the antioxidant functional group to the polymer backbone (Scheme 1) giving a macro-alkoxyamine or NO-R compound.

**Scheme 2:**
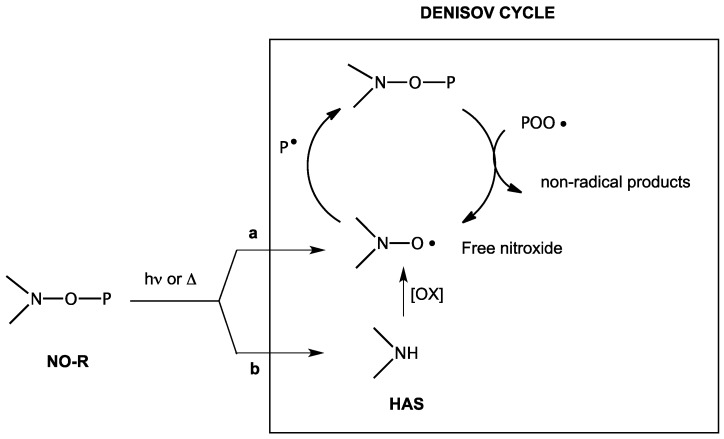
Role of NO-P derivative in the Denisov cycle. Route a: homolytic bond cleavage of the NO-P bond and Route b: homolytic bond cleavage of the N-OP bond. P is the polymer chain.

Since macro-alkoxyamines can be considered intermediates in the Denisov cycle of Hindered Amine Stabilizer (HAS), also the NO-P bond (where P is the polymer chain) generated by the grafting of BHB-T onto EOC can potentially exhibit an antioxidant activity in agreement with the reactions reported in Scheme 2 [32,33,34,35]. Moreover, the simultaneous presence of the hindered phenol moiety and of the NO-P group both immobilized onto the same polymer matrix suggests that these two units can favorably interact with each other in a synergistic mechanism for the stabilization of the polymer matrix [35,36,37,38].

In detail, in this paper the antioxidant role of the grafted BHB-T was evaluated both under photo- and thermo-oxidation conditions by carrying out molecular weight measurements, FT-IR analysis, Oxidation Induction Time (OIT) and Oxidation Onset Temperature (OOT) determinations. Special attention was given to verify if the NOP functionality can play some role in the antioxidant stabilization under accelerated oxidation experiments. Moreover, to point out the relevance of the immobilization of an AO onto a polymer matrix some comparison tests were also made by analyzing polymer samples containing free AOs. Finally, some explorative migration tests were used to simulate a possible application of the materials prepared in this paper. 

## 2. Materials and Methods

### 2.1. Materials

4-Hydroxy-2,2,6,6-tetramethylpiperidine-1-oxyl (HO-T) (Fisher Scientific Italia, Milan, Italy), 4-benzoyloxy-2,2,6,6-tetramethyl-piperidine-1-oxyl (Bz-T) (Fisher Scientific Italia), 3,5-di-*tert*-butyl-4-hydroxybenzoic acid (Fisher Scientific Italia), thionyl chloride (Fisher Scientific Italia), triethylamine (Fisher Scientific Italia), di(*tert*-butylperoxy-isopropyl) benzene (mixture of isomers) (P14) (Perkadox 14S-FL, Akzo Nobel, Amsterdam, The Netherlands) and 3,5-di-*tert*-butyl-4-hydroxybenzoic acid hexadecyl ester (CY, Cyasorb 2908 kindly supplied by Prof. Cristian Gambarotti, University of Milano, Milan, Italy) were used as received. A random copolymer ethylene/α-olefin (EOC) having density = 0.9 g/cm^3^ and *M*_n_ ≈ 90,000 D was used as polymer matrix. EOC has melting point between 50 and 70 °C [39] and *T*_onset_ about 430 °C (TGA analysis carried out under nitrogen) [25]. Pre-coated aluminum F254 silica gel 60 sheets were used for TLC analyses. Purification by chromatography was performed using silica gel Merck 60 (particle size 0.040–0.063 mm) (Merck KGaA, Darmstadt, Germany). All reactions were performed under nitrogen. The sample EOC-*g*-(Bz-T) where 4-benzoyloxy-2,2,6,6-tetramethyl-piperidine-1-oxyl (Bz-T) is grafted to EOC was prepared as previously reported [22].

### 2.2. Samples Preparation

#### 2.2.1. Synthesis of 3,5-di-*tert*-butyl-4-hydroxybenzoyl-2,2,6,6-tetramethylpiperidine-1-oxyl (BHB-T)

**Scheme 3:**
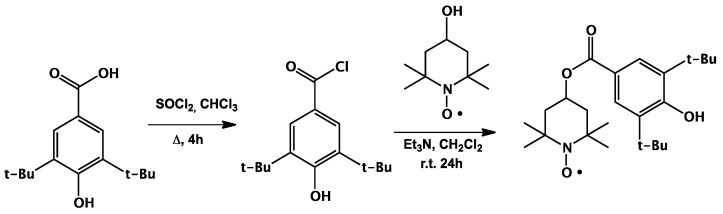
Synthesis of 3,5-di-*tert*-butyl-4-hydroxybenzoyl-2,2,6,6-tetramethylpiperidine-1-oxyl(BHB-T).

The preparation of BHB-T was carried out by a two-step procedure without isolation and purification of the intermediate product (Scheme 3) [40].

To a solution of 3,5-di-*tert*-butyl-4-hydroxybenzoic acid (4.00 g, 0.016 mol) in chloroform (40 mL), 4.76 g (0.04 mol) of thionyl chloride were added, under stirring. The obtained solution was stirred under reflux for 4 h. At the end of the reaction, the solvent and the excess of thionyl chloride were removed under vacuum and the crude 3,5-di-*tert*-butyl-4-hydroxybenzoyl chloride was used without further purification by dissolving the product with 40 mL of dichloromethane [41]. To the solution, HO-T (2.76 g, 0.016 mol) and after the dissolution of the reagents, triethylamine (2.45 mL, 0.017 mol) were added at room temperature. The resulting solution was stirred at room temperature for 24 h. Crude mixture was hydrolyzed with water (50 mL) and extracted with dichloromethane (3 × 50 mL). BHB-T was purified by column chromatography by using dichloromethane/ethyl acetate (95/5) as eluting mixture. Yield 50%. MS (CI): *m*/*z*: 427 [M + 23], 405 [M + 1], 404 [M]; FT-IR (KBr): *ν* = 3548, 2957, 1712, 1599, 1462, 1434, 1365, 1313, 1228, 1131, 986, 771 cm^−1^. C_24_H_38_NO_4_ Calculated C, 71.25; H, 9.47; N, 3.46. Found: C, 71.40; H, 9.50; N, 3.49. Melting point: 170 °C (lit. 174–176 °C [40]).

#### 2.2.2. Polymer Samples Preparation

Polymer samples were prepared in the melt by using an internal batch mixer (Brabender Plastograph OHG47055, Brabender^®^ GmbH & Co., KG, Duisburg, Germany) with a chamber of 30 mL. Torque and temperature data were acquired by Brabender Mixing software Win-Mix ver.1.0.

Functionalization of EOC was carried out at 170 °C, 50 rpm for 20 min. 20 g of EOC were introduced in the hot mixer and after its melting BHB-T and one minute later P14 were introduced in the mixer chamber (Table 1). During the melt mixing of the polymer in the Brabender chamber, the temperature of the molten polymer was higher than that set because of the shear stresses as shown in Figure S1 (Supplementary Material) that reports the temperature and the torque behavior recoded during the functionalization of EOC with BHB-T. The profile shows that the temperature of the molten matrix is about 175–176 °C. In this condition, the melting and uniform distribution of BHB-T inside the polymer is guaranteed. Moreover, the FD, determined on replicates shows good uniformity in the grafting distribution (see the values of standard deviation reported in Table 1). Functionalized samples were cut in small pieces and extracted with boiling acetone for 16 h and further purified by dissolution in hot toluene and precipitation from acetone. Samples were dried to constant weight and analyzed. Despite the double purification method, a small amount of free nitroxide can be usually detected even after purification (about 5–10 mol % of the amount evaluated by FT-IR after purification). 

The sample EOC/CY_2 was prepared in the melt by adding Cyasorb 2908 (CY) (0.09 mol %) to melted EOC at 170 °C (50 rpm, 10 min) (Table 2).

The samples EOC/(BHB-T)_2 and EOC/(Bz-T)_2 were prepared in solution by dissolving 500 mg of EOC in 10 mL of toluene at 90 °C. After the dissolution of the polymer, the solution was cooled to room temperature and 0.09 mol % of BHB-T or Bz-T were added (Table 2). The mixture was stirred at room temperature for 30 min and dried under reduced pressure to constant weight.

The functionalization degree (FD) of EOC-*g*-(BHB-T) samples, expressed as the mole of grafted nitroxide per 100 moles of monomer repeating units of polymer, was evaluated by FT-IR analysis by preparation of an opportune calibration curve that was obtained by adding a known amount of BHB-T to a toluene solution of EOC. FT-IR spectra were collected onto films obtained by solution casting of the EOC solutions containing different amount of BHB-T. The ratio between the area of the signal at 1715 cm^−1^ (carbonyl stretching of BHB-T) and of the band at 720 cm^−1^ (methylene rocking of polyethylene) used as internal reference, was plotted versus the amount of BHB-T. The calibration curve was obtained by the linear fitting of the data (Figure S2, Supplementary Material) and was used to evaluate the FD of functionalized EOC samples (Table 1). The FD of EOC-*g*-(Bz-T) was evaluated as previously reported [22].

### 2.3. Characterization

FT-IR spectra were recorded by the Fourier Transform Spectrometer Perkin Elmer Spectrum 100 (Waltham, MA, USA). Spectra of microcrystalline samples were obtained by potassium bromide pellet technique. Spectra of polymers were obtained on films prepared by compression molding at 110 °C under a pressure of about 6 tons by using the Carver press 3851-0 (Carver, Inc., Wabash, IN, USA). For the deconvolution of FT-IR spectra, six Gaussian shaped bands were considered indicatively at 1700, 1710, 1720, 1735, 1750, and 1780 cm^−1^ by using the non-linear curve fitting analysis of Origin 7.5. Polymer films for OIT and OOT analysis were obtained at 110 °C, at 1 ton for 1 min, film thickness 100 μm.

Melting point was determined by a MEL-TEMP^®^ capillary melting point apparatus (Fisher Scientific Italia).

Thermo gravimetric analyses (TGA) were carried out with the instrument EXSTAR 7200 TGA/DTA (Hitachi High-Tech Science Corporation, Tokyo, Japan). In a typical experiment, the sample (about 10 mg) was placed in an alumina sample pan and the run was carried out at a standard rate of 10 °C/min from 30 to 700 °C under nitrogen flow. BHB-T has *T*_onset_ = 267 °C, CY has *T*_onset_ = 257 °C, under nitrogen. Thermograms of EOC-*g*-(Bz-T) and EOC-*g*-(BHB-T)_2 show two main weight losses (Figure S3, Supplementary Material), the first, associated to the detaching of the TEMPO moiety [25,26] has *T*_onset_ = 245 °C in the case of EOC-*g*-(Bz-T) and *T*_onset_ = 250 °C in the case of EOC-*g*-(BHB-T)_2. The second weight loss is due to the decomposition of the polymer matrix and occurs at about 430 °C.

The photo-oxidation was performed on polymer pellets. EOC, EOC-*g*-(Bz-T) and EOC-*g*-(BHB-T)_2 were used. Photo-oxidative degradation of samples was carried out on a QUV PANEL apparatus (Q-LAB, Cleveland, OH, USA) at 60 °C with continued exposure to UV radiation up to 20 days. At least two separate samples were analyzed at each exposure time. The irradiance of the UV lamps (UVA 340 lamps) is 0.68 W/m^2^ and has a broad band with a maximum at 340 nm. Photo-oxidized pellets were removed from the UVA source and subsequently added to appropriate amount of solvent (methanol/acetonitrile 90:10) to extract degradation products. At each exposure time at least two replicate samples were analyzed by electrospray ionization ESI. All samples injections were performed 3 h after the addition of solvent mixture in order to wait the necessary time for the extraction of photo-exposed products. 

ESI mass spectra of photo-oxidized samples were acquired by a Thermo Scientific Exactive Plus Orbitrap MS (Thermo Fischer Scientific, San Jose, CA, USA), using a heated electrospray ionization (HESI II) interface. Mass spectra were recorded operating in positive ion mode in the *m*/*z* range 120–500 at a resolving power of 25,000 (full-width-at-half-maximum, at *m*/*z* 200, RFWHM), resulting in a scan rate of ≥2.5 scans/s when using automatic gain control target of 1.0 × 10^6^ and a C-trap inject time of 100 ms under the following conditions: capillary temperature 200 °C, nebulizer gas (nitrogen) with a flow rate of 30 arbitrary units; auxiliary gas flow rate of 10 arbitrary units; source voltage 5 kV; capillary voltage 82.5 V; tube lens voltage 85 V. 1 μL of each sample was injected with auto sampler into the mass spectrometer, using methanol/acetonitrile (90:10, *v*/*v*) as solvent at a flow rate of 100 μL/min. The Orbitrap MS system was tuned and calibrated in positive modes, by infusion of solutions of a standard mixture of caffeine (Mr 194.1 Da), MRFA peptide (Mr 423.6 Da) and Ultramark (Mr 1621 Da). Data acquisition and analysis were performed using the Excalibur software.

Molecular weight distribution (MWD), intrinsic viscosity (η) and Mark-Houwink-Sakurada (MHS) plot of the polymers were obtained by a high temperature multi-detectors size exclusion chromatography (SEC) GPCV2000 system from Waters (Waters Corporation, Milford, MA, USA). The High Temperature Size Exclusion Chromatography (HT-SEC) chromatographic system was equipped with two on-line detectors: a differential viscometer (DV) and a differential refractometer (DRI) as concentration detector. The running SEC-DV experimental conditions were the following: three HT columns (806M-806M-803) from Shodex (Showa Denko America, Inc., New York, NY, USA), ortodichlorobenzene (ODCB) as mobile phase, 0.8 mL/min of flow rate and 145 °C of temperature. For the EOC-*g*-(Bz-T) samples a little different SEC column set (Shodex HT 806M-806M-805) was used; however the quantification of the molecular weight variations during photo-oxidation were meaningful. The universal calibration was constructed using 18 polystyrene (PS) standards with narrow MWD and peak molecular weight (*M*_p_) ranging from 5480 kg/mol to 162 g/mol. The SEC-DV system was described in detail elsewhere [42]. Polymer concentration was about 2.0 mg/mL in ODCB solvent stabilized with an antioxidant (Ionol 0.05%). After complete dissolution (1 h at 160 °C in oven) solutions were filtered through 0.5 μm filter and then kept at 145 °C before injection.

Thermal oxidation was carried out on polymer films (30 μm thick) at 110 °C in a static oven. FT-IR spectra were periodically collected and Carbonyl Index (CI) was calculated as the ratio of the areas of the carbonyl absorption region between 1850 and 1600 cm^−1^ to that of a polymer reference peak between 1980 and 2110 cm^−1^. 

The Oxidation Induction Time (OIT) and the Oxidation Onset Temperature (OOT) were determined by using a Perkin-Elmer DSC-4000 differential scanning calorimeter thermal analyzer equipped with a 3 stage cooler able to reach −130°C. The instrument was calibrated by using indium (m.p. 156.6 °C, Δ*H* = 28.5 J/g) and zinc (m.p. 419.5 °C). OIT measurements were carried out according to following method: step (1) 30 °C under nitrogen flow (50 mL/min) for 5 min; step (2) 30–190 °C under nitrogen flow (50 mL/min) at 10 °C/min; step (3) hold at 190 °C under nitrogen flow (50 mL/min) for 5 min; step (4) at 190 °C switch to oxygen flow (50 mL/min), hold for 60 min. The oxidation of the sample was observed as a sharp increase in heat flow due to the exothermic nature of the oxidation reaction. The OIT value was obtained by curve elaboration carried out by the software Pyris 9 (Perkin Elmer). The values reported in Table 3 and Table 4 are the average of three measurements. The OOT measurements were carried out by heating (10 °C/min) the sample (about 5 mg) in an open aluminum pan under oxygen flow (50 mL/min). The oxidation of the sample was observed as a sharp increase in heat flow due to the exothermic nature of the oxidation reaction. The OOT represents the onset temperature of the exothermic peak and was obtained by the software Pyris 9 (Perkin Elmer). The values reported in Table 2 and Table 4 are the average of three measurements.

Migration analyses were carried out on films of EOC-*g*-(BHB-T)_2 and EOC/CY_2 that were obtained by a Carver laboratory press between two Teflon sheets, at 150 °C, followed by cooling at ambient temperature. Films, of about 200 μm thick, were put in contact with a fixed volume of ethanol. The release kinetics of the BHB-T and CY were performed by ultraviolet spectrometric measurements at ambient temperature, using a Spectrometer UV-2401 PC Shimadzu (Kyoto, Japan). The tests were performed using rectangular specimens of 4 cm^2^ and same thickness (200 μm), placed into 20 mL of ethanol and stirred at 100 rpm in an orbital shaker (VDRL MOD. 711+, Asal S.r.l., Milan, Italy). The release medium was withdrawn at fixed time intervals and replenished with fresh medium. A UV-Vis calibration curve was obtained for EOC in ethanol (Figure S4, Supplementary Material). The considered band was at 264 nm.

## 3. Results and Discussion

### 3.1. Preparation and Structural Characterization of the Samples

**Figure 1:**
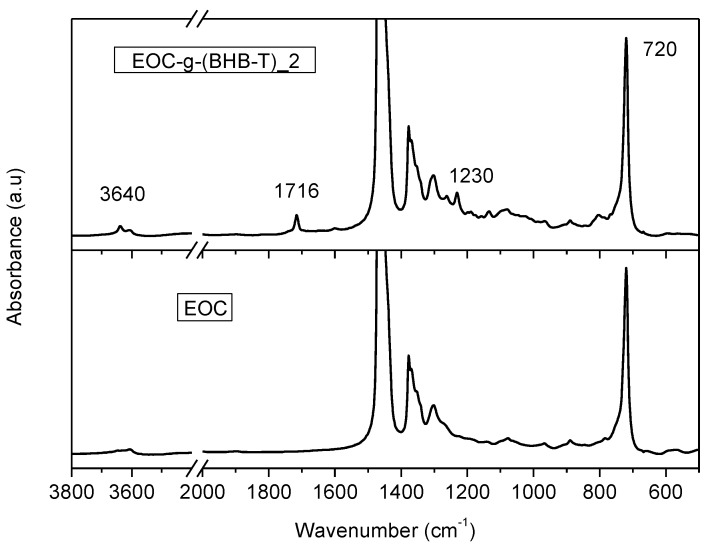
Comparison between the FT-IR spectra of EOC and EOC-*g*-(BHB-T)_2.

BHB-T was previously described in the literature as a possible precursor of a di-radical species, but its preparation was only roughly reported [40] and its possible antioxidant ability was not discussed. Accordingly, BHB-T was here prepared by esterification of 3,5-di-*tert*-butyl-4-hydroxybenzoic acid with 4-hydroxy-2,2,6,6-tetramethylpiperidine-1-oxyl (HO-T) as reported in the Section 2 (Scheme 3). The product was characterized by mass spectrometry, FT-IR (Figure S5, Supplementary Material) and TGA under nitrogen (*T*_onset_ under nitrogen = 267 °C). The TEMPO moiety of the BHB-T was grafted to EOC by NRC reaction as previously described [20,22,24]. Two samples were prepared at different feed conditions (Section 2, Table 1). The FT-IR spectra of the purified samples, compared with that of the pristine EOC (Figure 1), evidenced the presence of a band at 1715 cm^−1^ attributable to the C=O stretching of BHB-T and a band at 3640 cm^−1^ due to the stretching of the OH group of BHB-T [12].

The functionalization degree (moles of grafted functional group per 100 moles of monomeric repeating unit) of the two samples was evaluated by FT-IR analysis through the preparation of an opportune calibration curve (see the Section 2, Table 1 and the Supplementary Material Figure S2). Results show that the amount of grafted BHB-T (about 0.5 and 1 wt. %) is consistent with the amount of AO usually added to stabilize polyolefins [43,44].

### 3.2. Photo- and Thermo-Oxidation Behavior of Functionalized EOC

#### 3.2.1. Photo-Oxidation Tests

The sample EOC-*g*-(BHB-T) 2 was photo-oxidized for different times by using an UV lamp emitting at 340 nm (see Section 2). For comparison purpose, also an EOC sample functionalized with 4-benzoyloxy-2,2,6,6-tetramethyl piperidine-1-oxyl (EOC-*g*-(Bz-T)) [22] was photo-oxidized under the same experimental conditions. The identification of the low molecular weight compounds produced and released during the degradation process is of remarkable importance to understand the possible mechanism accounting for deterioration and/or stabilization of polymer materials [45]. Therefore, low molecular weight species formed during photo-exposure were extracted with a methanol/acetonitrile mixture and analyzed by electrospray ionization-mass spectroscopy (ESI-MS) in positive ion mode so only positive polarized products were identified.

**Figure 2:**
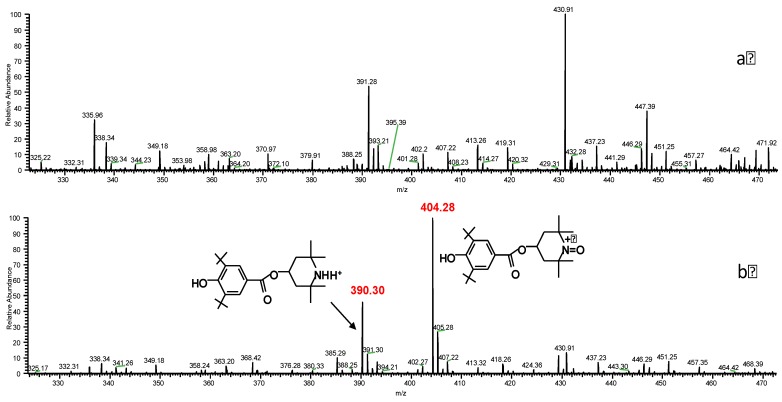
Electrospray ionization-mass spectroscopy (ESI-MS) registered in positive mode, in the mass range 300–480 *m*/*z*, of the products extracted from EOC-*g*-(BHB-T)_2 virgin sample (**a**) and photo-oxidized for 6 days (**b**).

In Figure 2, ESI-MS spectra of the products extracted from EOC-*g*-(BHB-T)_2 before and after 6 days of photo-oxidation are reported.

Before irradiation, the ESI mass spectrum of the products extracted from EOC-*g*-(BHB-T)_2 (Figure 2a) shows peaks that can be assigned to some impurities present in the solvent/background signal and/or to some additives of the polymer, but no signals attributable to BHB-T or its derivatives were detected. After 6 days of photo-exposure, the signal at *m*/*z* 404.28, belonging to BHB-T, and a peak at *m*/*z* 390.30 reasonably assigned to the parent amine of BHB-T, appeared [32,46,47] (Figure 2b). Similar results were obtained in the case of EOC-*g*-(Bz-T): the ESI mass spectra of this sample before and after photo-exposure are reported in the Supplementary Material (Figure S6). The presence of these species all over the irradiation period (20 days) pointed out a prolonged action of both grafted TEMPO moieties that is likely due to the presence of a covalent bond between TEMPO functionality and polymer matrix.

**Figure 3:**
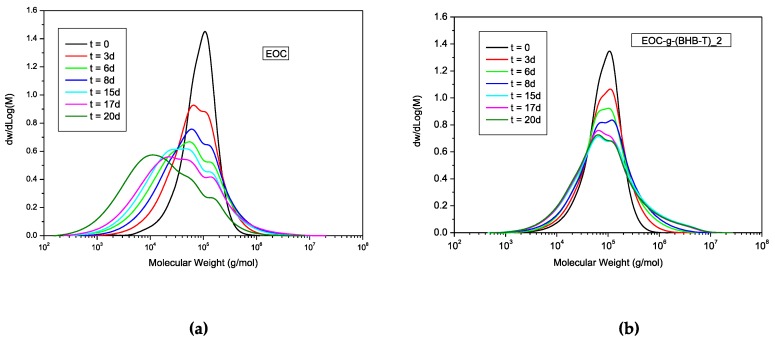
Differential molecular weight distribution (MWD) of EOC (**a**) and EOC-*g*-(BHB-T)_2 (**b**) before and after different UV irradiation times.

The photo-exposed polymer samples were then analyzed by HT-SEC with the aim to evaluate the molecular weight variation during irradiation (Figure 3 and Figure S7 and Table S1 in Supplementary Material).

In the case of EOC (Figure 3a), the photo-oxidation caused a large modification of the pristine molecular structure of the polymer as evidenced by the presence of long tails at high molecular weights, especially for low irradiation time, and by a general shift of the curves towards low molecular weights. In the case of EOC-*g*-(BHB-T)_2 (Figure 3b), although a large and partially multimodal molecular weight distribution was observed, especially for long irradiation time, a greater control of the molecular structure was obtained. In particular, both the *M*_n_ and the apparent viscosity, related to the hydrodynamic radius of macromolecules, (Figures S8 and S9 and Table S1 in Supplementary Material) were subjected to a very limited variation during the irradiation process.

**Figure 4:**
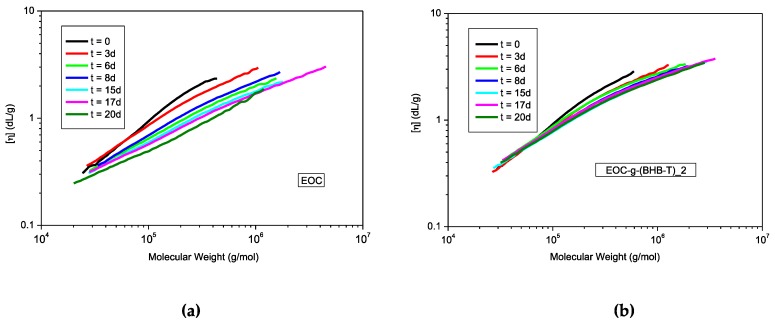
Mark-Houwink-Sakurada (MHS) plot before and during photo-oxidation of EOC (**a**) and EOC-*g*-(BHB-T)_2 (**b**).

Further evidences of the photo-stability of EOC-*g*-(BHB-T)_2 with respect to EOC can be obtained by analyzing the Mark-Houwink-Sakurada (MHS) plots (Figure 4) before and during irradiation. Indeed, in the case of EOC-*g*-(BHB-T)_2 (Figure 4b), all curves belonging to irradiated samples deviate from linearity and also from the curve of the sample at *t* = 0. This is particularly evident for prolonged irradiation time and in the region of high molecular weights. This deviation is especially observed for polymers having long chain branching [48] and suggests that during the photo-oxidation of EOC-*g*-(BHB-T)_2 branching was the main phenomenon. Instead, in the case of EOC (Figure 4a), a translation of the whole curve of the photo-oxidized EOC samples with respect to that of the pristine polymer was observed also for short irradiation time. This behavior suggests that chain breaking was occurring during the early stage of oxidation. 

Finally, HT-SEC analysis of EOC-*g*-(Bz-T) (Figure S7 and Table S1 in the Supplementary Material) evidenced that a large variation of the macromolecular structure, mainly due to chain scission, was provided for long irradiation times, but the small variation of both *M*_n_ and intrinsic viscosity (Figures S8 and S9, Supplementary Material) observed during the first period of photo-exposition suggests that also the grafted TEMPO moiety could have a limited stabilization effect probably related to the NO-P functionality.

#### 3.2.2. Thermo-Oxidation Tests

The antioxidant stabilization ability of the hindered phenol moiety of BHB-T as well as that of the NO-P bond formed by covalent grafting between the TEMPO unit and polymer chain is generally discussed in terms of photo- rather than thermo-oxidation stabilization. However, some results about the possible use of HAS [34,36,37], HAS-NOR [34,37], and p-hydroxybenzoate [49] as stabilizers under thermo-oxidation conditions have been discussed in the literature. So with the aim to establish the stabilization ability of the p-hydroxybenzoate moiety of BHB-T and to state the role of the NO-P bond under thermo-oxidation conditions, some tests were carried out by analyzing EOC functionalized with BHB-T or Bz-T. Moreover, to compare the antioxidant activity of free and bonded AOs, two physical blends between EOC and the 3,5-di-*tert*-butyl-4-hydroxybenzoic acid hexadecyl ester (CY), used as a free AO model of BHB-T, or the free BHB-T were analyzed.

**Figure 5:**
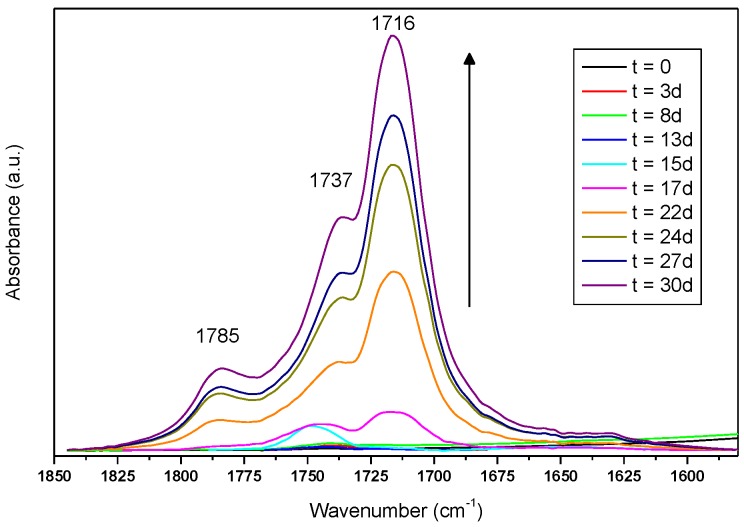
FT-IR spectra of EOC collected during oxidation process. The spectra were normalized to the band at 720 cm^−1^ (methylene rocking) characteristic of the polymer matrix.

Accelerated oxidation was carried out at 110 °C in an oven and was followed by FT-IR spectroscopy by periodically recording the spectra of the polymer samples. The temperature chosen for the experiment is over that of EOC melting (50–60 °C) [39] to speed up the process. Superimposition of the FT-IR spectra clearly evidenced that the thermal oxidation of the pristine polymer matrix (Figure 5) gives rise to the formation of different oxidation products. In agreement with the literature [50], the observed bands can be attributed to the formation of conjugated ketone (1700 cm^−1^), carboxylic acid (1712 cm^−1^), ketone (1721 cm^−1^), ester (1737 cm^−1^), peroxy acid or ester (1755 cm^−1^), and lactone (1783 cm^−1^) groups (as compared with Figure S10 that reports the deconvolution of the spectrum recorded after 30 days of thermal oxidation). 

**Figure 6:**
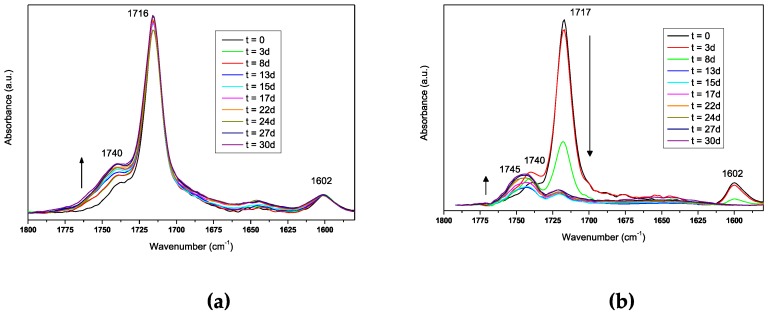
FT-IR spectra of EOC-*g*-(BHB-T)_2 (**a**) and EOC/(BHB-T)_2 (**b**) collected during the thermo-oxidation process. The spectra were normalized to the band at 720 cm^−1^ (methylene rocking).

**Figure 7:**
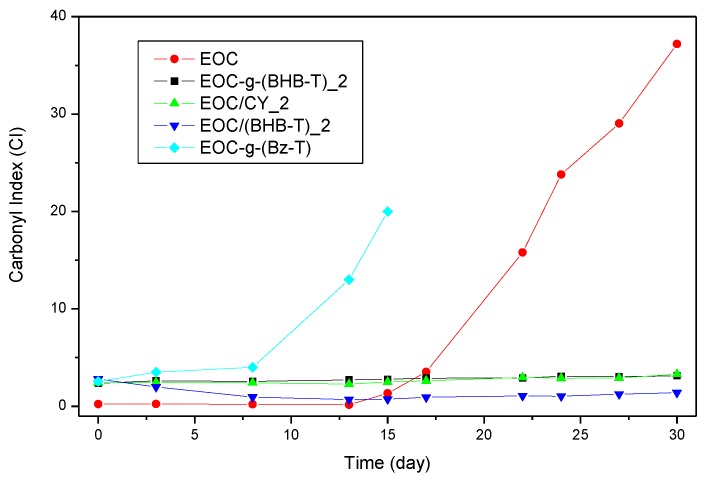
Carbonyl Index (CI) evolution vs. oxidation time.

In the case of the functionalized EOC-*g*-(BHB-T)_2 (Figure 6a) as well as for the analogous physical blend EOC/(BHB-T)_2 (Figure 6b), the spectra collected during oxidation showed that the presence of bonded or free AO confers a large control of the polymer oxidation as evidenced also by the Carbonyl Index (CI) evolution as a function of oxidation time (Figure 7). 

However, some differences between the free and bonded AO can be observed; indeed, in the case of EOC-*g*-(BHB-T)_2 (Figure 6a), where the antioxidant is bonded to the polymer chain, the intensity of the main signal of the grafted functional group at 1716 cm^−1^ as well as that of the band at 1602 cm^−1^, which is due to the aromatic ring stretching of the grafted BHB-T, did not change. Only a small signal at about 1740 cm^−1^, probably due to ester vibration, that is present also at t = 0, increased during oxidation (Figure 6a); no other signals attributable to the polymer oxidation were observed. Whereas, when BHB-T is mixed to EOC in the sample EOC/(BHB-T)_2 (Figure 6b), the thermal oxidation caused first the decrease of both the signals at 1717 cm^−1^ and at 1602 cm^−1^ followed by the increase of a band at 1745 cm^−1^ that can be attributed to a preliminary oxidation of the polymer matrix. Notably, the signal at 1740 cm^−1^ is present also in this sample and it was visible from the beginning of the experiment.

**Figure 8:**
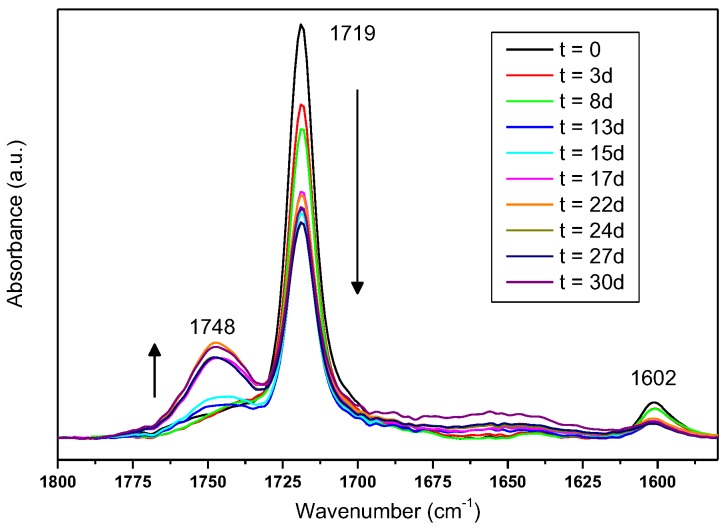
FT-IR spectra of EOC/(CY)_2 collected during the thermo-oxidation process. The spectra were normalized to the band at 720 cm^−1^ (methylene rocking).

Similarly, in the case of EOC/CY_2, where CY is free, the carbonyl signal as well as the band at 1602 cm^−1^ both attributable to the presence of CY, decreased during oxidation and a new signal at about 1748 cm^−1^ increased (Figure 8). These results suggest that although free AO molecules can preserve the polymer from oxidation, their mobility can cause the migration and the partial loss of the functional molecules as evidenced by the decrease of their characteristic signals. This occurs especially in the case of BHB-T that has a lower molecular weight than CY and probably also a limited affinity for the polymer matrix.

Finally, collected data showed that grafted Bz-T seems not to be able to preserve the polymer matrix from oxidation; indeed, FT-IR spectra of EOC-*g*-(Bz-T), where Bz-T is bonded to the polymer matrix, evidenced the oxidation of the polymer matrix in less than 7 days (Figure 7). 

Thermal oxidation behavior of EOC samples was also investigated by determining OIT and OOT by DSC analysis (Table 3) [51,52]. Both methods are used also in the industry for estimating the resistance of a polymer under severe oxidation conditions and it is a quick tool to determine the stability of materials against thermally induced oxidation, especially when materials having low melting temperature are used. 

In details, OIT represents the time that elapses before the sample exhibits an exothermic oxidation reaction in an isothermal experiment, whereas OOT corresponds to the onset temperature of the exothermal curve registered under dynamic experimental conditions by gradually increasing the temperature of the sample under oxygen flow [52]. 

OIT results, collected at 190 °C (Figure S11, Supplementary Material), as well as OOT values recorded for the samples that have the functional group immobilized onto the polymer matrix (Table 3) evidenced that BHB-T functionalized samples showed both longer OIT times and higher OOT temperatures than pristine polymer suggesting that the hindered phenol unit can effectively protect the polymer. Moreover, longer OIT and higher OOT values were observed in the case of the sample having the highest FD. The literature documents that in some cases a linear correlation between the amount of AO and the increase of OIT values can be observed [53]. On the contrary, the presence of grafted Bz-T does not guarantee a significant oxidative resistance of the polymer matrix, confirming previous results from accelerated oxidation testing. 

Interestingly, the OIT and OOT data collected from the samples where the additives are not bonded (Table 4) showed that the free molecules provided higher thermal oxidation stability to the polymer matrix than the corresponding immobilized form (Compare data in Table 3 and Table 4). Probably two main effects can be claimed to explain these results: the first one is the different mobility of the AO inside the matrix indeed AOs that are free to migrate and to diffuse near the surface of the polymer, where the oxidation process should start [35,54], are reported to be more efficient in the stabilization of the polymer than immobilized AOs [55]. The latter is ascribable to the action of the free nitroxide functionality that in the second set of experiments is not bonded to the polymer and thus can take part directly to the Denisov cycle (Scheme 2). Indeed, the HAS-NOR stabilizing action was reported to depend on the formation of the free nitroxide that is considered the key species for the protection against oxidation processes [31], Scheme 2. However, as reported in the literature, the formation of free nitroxide from HAS-NOR compounds where the TEMPO moiety is involved in a covalent bond with a polymer matrix is not favored [33,35,38]. Another possible cause of the lower OIT and OOT values measured from the EOC sample with bonded BHB-T with respect to EOC sample with free BHB-T, can be the partial “sacrificial” loss of the hindered phenol moiety due to its interference with free radical functionalization process. Actually, literature reports that hindered phenol antioxidant can react with the radical species that is formed during the reaction of polyolefins with peroxide causing a decrease of the AO concentration and of its availability at the end of the process [56,57]. However, p-hydroxybenzoate antioxidants (like BHB-T) are less reactive than other hindered phenol molecules so probably their reaction with radical species formed during the functionalization of EOC is limited. Moreover, the differences in the OIT data reported in Table 3 and Table 4 are too high to totally attribute this difference to the consumption of the antioxidant during the functionalization.

## 4. Migration Tests

**Figure 9:**
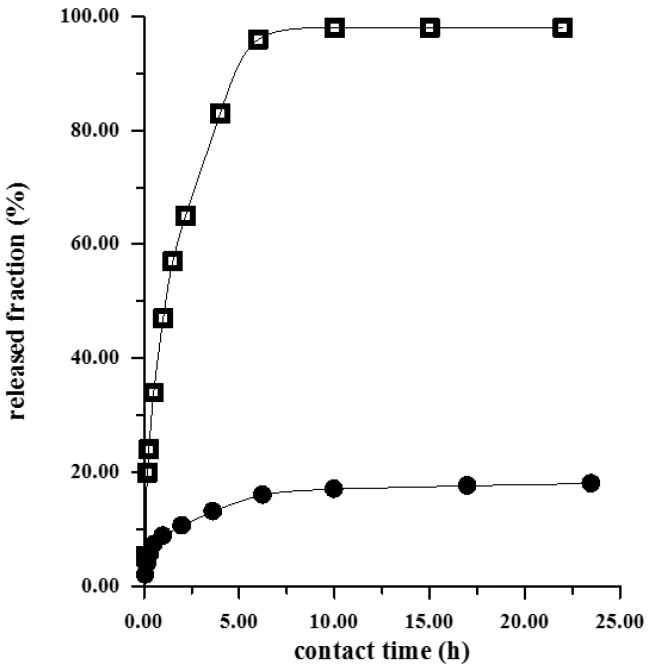
Relative fraction of extracted antioxidant (AO) as a function of contact time (h) with ethanol for samples: (•) EOC-*g*-(BHB-T)_2; (□) EOC/CY_2.

Although some of the results previously discussed evidenced that free AOs, under some experimental conditions, work better than bonded AOs, the main aim of the paper is to underline the relevance of the immobilization of an AO to prolong the service life of a polymeric product and to demonstrate that even after the contact of functionalized EOC with possible extracting solutions, the AO activity of the grafted BHB-T still persists. To test the effectiveness of the BHB-T immobilization, migration tests were carried out. Accordingly, EOC-*g*-(BHB-T)_2 film was suspended in ethanol and periodically UV-Vis spectra of the ethanol phase were recorded. From the UV-Vis analysis and by FT-IR determination it was observed that only about 18 wt. % of BHB-T was extracted from the EOC-*g*-(BHB-T)_2 film. For comparison, migration test was carried out also on the sample EOC/CY_2 where CY is only mixed to EOC, in this case a complete migration of CY was observed (Figure 9). 

To exclude that the differences observed in terms of extracted amount for the two molecules can be due to their different migration ability (Figure S12, Supplementary Material), the diffusion coefficient *D* was calculated by using the Equation (1) [58]:

*Ct/Ceq* = 4/*d*(*Dt*/*π*)^1/2^ (1)

where *Ct* is the concentration of the molecule at time t into the solution and *Ceq* is the concentration of the molecule at equilibrium, *d* (cm) is the sample thickness. By applying this equation the diffusion coefficient resulted to be 2.10 × 10^−8^ cm^2^/s for both samples. The fact that both samples showed a Fickian behavior regarding the diffusion of CY and of the free BHB-T, confirms that the different concentration detected in solution is not related to different migration kinetics.

To further prove that the contact with ethanol completely remove the mixed CY whereas only a small fraction of BHB-T was removed, OIT and OOT analysis were repeated after migration test (Table 5). 

As expected on the basis of data reported in Figure 9, in the case of EOC-*g*-(BHB-T)_2_EX the contact with ethanol caused a small decrease of OIT and OOT values, whereas in the case of EOC/CY_2_EX, OIT and OOT values are very similar to that of the starting polymer suggesting a complete loss of CY that was extracted from the polymer matrix.

## 5. Conclusions

A bi-functional molecule (BHB-T) consisting of a TEMPO moiety and a hindered phenol group covalently connected was successful synthesized. The TEMPO functionality was exploited to graft BHB-T onto a copolymer ethylene/α-olefin (EOC) by NRC reaction, following a method that allows the polymer to maintain its structure by modulating and controlling the reaction conditions. 

From the data so far discussed, it can be concluded that, independently from the oxidation conditions and by combining the OIT-DSC measurements, the results collected from the accelerated thermo-oxidation in the oven and the HT-SEC data of photo-oxidized samples, the antioxidant activity of BHB-T grafted to EOC is mainly played by the p-hydroxybenzoate moiety rather than by NO-P group. Indeed, when Bz-T is grafted to EOC no stabilization of the polymer matrix under thermo-oxidation was observed. This suggests that the bond between Bz-T and EOC is so strong that is hardly broken under oxidative conditions thus not permitting Denisov cycle. However, under photo-oxidation conditions, the formation of the free nitroxide and of the parent amine of Bz-T and BHB-T from functionalized EOC samples, suggests that both grafted TEMPO derivatives can be potentially active as an AO in the stabilization mechanism. 

Finally, migration tests, carried out by suspending films of functionalized EOC-*g*-(BHB-T) and EOC/CY physical blend in ethanol and then collecting UV-Vis spectra of the ethanol phase at different time, evidenced a complete migration of the CY, whereas only a small amount of BHB-T migrated out of the functionalized polymer and it was demonstrated that the thermal oxidative stability of this sample was mostly retained. Moreover, under relatively low temperature oxidation conditions and long times of execution, as in the case of accelerated oxidation tests, the immobilization of the AO has some advantages because it improves polymer thermo-oxidative stability and avoids migration of AO species, respectively.

## Figures and Tables

**Table 1:** Functionalized polymer samples: feed composition and functionalization degree.

Sample name	Feed composition		Functionalization degree (FD) ^1^ (mol %)
	Functional molecule (mol %)	Peroxide (mol %)	
EOC-*g*-(BHB-T)_1	BHB-T 0.14)	0.04	0.05 ± 0.01
EOC-*g*-(BHB-T)_2	BHB-T (0.28)	0.08	0.09 ± 0.01
EOC-*g*-(Bz-T) [22]	Bz-T (0.50)	0.21	0.23 ± 0.03

^1^ Functionalization Degree (FD): moles of the grafted functional groups with respect to 100 moles of monomer repeating unit.

**Table 2:** Polymer mixtures: amount of mixed additives.

Sample name	Type and amount of free additive (mol %) ^1^
EOC/(BHB-T)_2	BHB-T (0.09)
EOC/CY_2	CY (0.09)
EOC/(Bz-T)_2	Bz-T (0.09)

^1^ Moles of added molecule with respect to 100 moles of monomer repeating unit.

**Table 3:** Oxidation Induction Time (OIT) and Oxidation Onset Temperature (OOT) values for EOC functionalized with Bz-T and BHB-T.

Sample	Amount of bonded functional group (mol %) ^1^	OIT (min) ^2^	OOT (°C) ^3^
EOC	-	<1	201.3 ± 0.5
EOC-*g*-(Bz-T)	0.23	<1	196 ± 1
EOC-*g*-(BHB-T)_1	0.05	10 ± 1	213 ± 1
EOC-*g*-(BHB-T)_2	0.09	16 ± 2	221 ± 1

^1^ The amount of functional group was expressed as moles of functional groups with respect to 100 moles of repeating monomeric units; ^2^ OIT recorded at 190 °C, is an average value of three repeating measurements; ^3^ OOT is an average value of three repeating measurements.

**Table 4:** Oxidation Induction Time (OIT) and Oxidation Onset Temperature (OOT) values for EOC mixed with BHB-T, Bz-T and CY.

Sample	Amount of mixed functional group (mol %) ^1^	OIT (min) ^2^	OOT (°C) ^3^
EOC/(BHB-T)_2	0.09	52 ± 7	239 ± 2
EOC/(Bz-T)_2	0.09	14 ± 3	231 ± 2
EOC/CY_2	0.09	50 ± 5	236.9 ± 0.3

^1^ The amount of functional group was expressed as moles of functional groups with respect to 100 moles of repeating monomeric units; ^2^ OIT recorded at 190 °C, is an average value of three repeating measurements; ^3^ OOT is an average value of three repeating measurements.

**Table 5:** OIT and OOT values after migration test.

Sample	Amount of functional group (mol %) ^1^	OIT (min) ^2^	OOT (°C) ^3^
EOC-*g*-(BHB-T)_2	0.09	16 ± 2	221 ± 1
EOC-*g*-(BHB-T)_2_EX ^4^	0.08	14 ± 2	205 ± 2
EOC/CY_2 ^5^	0.09	50 ± 5	236.9 ± 0.3
EOC/CY_2_EX ^4,5^	-	<1	181 ± 0.6

^1^ The amount of functional group (i.e., mole of functional groups with respect to 100 moles of repeating monomer units) was determined before and after migration test; ^2^ OIT recorded at 190 °C, average value of three measurements; ^3^ OOT, average value of three measurements; ^4^ The sample was analyzed after the migration test; ^5^ In this sample CY is mixed and not bonded to the polymer matrix.

## Supplementary Materials

The following are available online at www.mdpi.com/2073-4360/9/12/670/s1, Figure S1: Torque curve and temperature profile recorded during the functionalization of EOC with BHB-T, Figure S2: Calibration curve for the determination of the FD of EOC-*g*-(BHB-T) samples, Figure S3: TGA thermograms and their first derivative of EOC-*g*-(Bz-T) and EOC-*g*-(BHB-T)_2, Figure S4: Calibration curve for the determination of the amount of CY and BHB-T released from EOC/CY2 and EOC-*g*-(BHB-T)_2 during migration tests, Figure S5: FT-IR spectrum of 3,5-di-*tert*-butyl-4-hydroxybenzoyl-2,2,6,6-tetramethylpiperidine-1-oxyl radical (BHB-T), Figure S6: ESI mass spectra registered in positive mode, in the mass range 200–300 *m*/*z*, of the products extracted from EOC-*g*-(Bz-T) virgin sample (a) and photo-oxidized for 6 days (b), Figure S7: Differential MWD of EOC-*g*-(Bz-T) before and after different UV irradiation times, Figure S8: *M*_n_ variation as a function of irradiation time, Figure S9: Intrinsic viscosity variation as a function of irradiation time, Figure S10. Deconvoluted IR spectrum of EOC thermo-oxidized for 30 days in the absorption region of carbonyl group, Figure S11: Oxidation induction time (OIT) curves of pristine EOC, EOC functionalized with Bz-T and BHB-T and EOC mixed with CY. Acquisition temperature 190°C, oxygen flow 50 mL/min, Figure S12: *Ct*/*Ceq* vs. square root of time (h) of EOC in ethanol for samples: (•) EOC-*g*-(BHB-T)_2; (□) EOC/CY_2, Table S1: SEC-DV characterization of EOC, EOC-*g*-(Bz-T) and EOC-*g*-(BHB-T)_2 during photo-oxidation.

## References

[1] Billingham N.C. (2006). Degradation and Stabilization of Polymers. Materials Science and Technology.

[2] Gijsman P. (2012). Handbook of Environmental Degradation of Materials.

[3] Hawkins W.L. (2012). Polymer Degradation and Stabilization.

[4] Gao X., Hu G., Qian H.Z., Ding Y., Zhang S., Wang D., Yang M. (2007). Immobilization of antioxidant on nanosilica and the antioxidative behavior in low density polyethylene. Polymer.

[5] Chen J., Yang M.S., Zhang S.M. (2011). Immobilization of antioxidant on nanosilica and the aging resistance behavior in polypropylene. Compos. Part A.

[6] Lonkar S.P., Kutlu B., Leuteritz A., Heinrich G. (2013). Nanohybrids of phenolic antioxidant intercalated into MgAl-layered double hydroxide clay. Appl. Clay Sci..

[7] Lonkar S.P., Leuteritz A., Heinrich G. (2013). Antioxidant intercalated layered double hydroxides: A new multifunctional nanofiller for polymers. RSC Adv..

[8] Dintcheva N.T., Al-Malaika S., Arrigo R., Morici E. (2017). Novel strategic approach for the thermo- and photo-oxidative stabilization of polyolefin/clay nanocomposites. Polym. Degrad. Stab..

[9] Perez Amaro L., Cicogna F., Passaglia E., Morici E., Oberhauser W., Al-Malaika S., Dintcheva N.T., Coiai S. (2016). Thermo-oxidative stabilization of poly(lactic acid) with antioxidant intercalated layered double hydroxides. Polym. Degrad. Stab..

[10] Al-Malaika S., Scott G., Wirjosentono B. (1993). Mechanisms of antioxidant action: Polymer-bound hindered amines by reactive processing, Part III Effect of reactive antioxidant structure. Polym. Degrad. Stab..

[11] Al-Malaika S., Suharty N. (1995). Reactive processing of polymers: Mechanisms of grafting reactions of functional antioxidants on polyolefins in the presence of a coagent. Polym. Degrad. Stab..

[12] Manteghi A., Ahmadi S., Arabi H. (2016). Covalent immobilization of phenolic antioxidant on Ethylene copolymers: An efficient approach toward enhanced long-term stabilization of polypropylene. Polymer.

[13] Kim T.H., Lee N. (2003). Melt-Grafting of Maleimides Having Hindered Phenol Group onto Polypropylene. Bull. Korean Chem. Soc..

[14] Chinelatto M.A., Agnelli J.A.M., Canevarolo S.V. (2015). Synthesis and photostabilizing performance of a polymeric HALS based on 1,2,2,6,6-pentamethylpiperidine and vinyl acetate. Polímeros.

[15] Wilén C.-E., Auer M., Strandén J., Näsman J.H., Rotzinger B., Steinmann A., King R.E., Zweife H., Drewes R. (2000). Synthesis of Novel Hindered Amine Light Stabilizers (HALS) and Their Copolymerization with Ethylene or Propylene over Both Soluble and Supported Metallocene Catalyst Systems. Macromolecules.

[16] Xue B., Ogata K., Toyota A. (2007). Synthesis and radical scavenging ability of new polymers from sterically hindered phenol functionalized norbornene monomers via ROMP. Polymer.

[17] Stagnaro P., Mancini G., Piccinini A., Losio S., Sacchi M.C., Viglianisi C., Menichetti S., Adobati A., Limbo S. (2013). Novel Ethylene/Norbornene Copolymers as Nonreleasing Antioxidantsfor Food-Contact Polyolefinic Materials. J. Polym. Sci. B Polym. Phys..

[18] Beer S., Teasdale I., Brueggemann O. (2013). Immobilization of antioxidants via ADMET polymerization for enhanced long-term stabilization of polyolefins. Eur. Polym. J..

[19] Al-Malaika S., Ibrahim A.Q., Scott G. (1988). Mechanisms of antioxidant action: Photo-antioxidant activity of polymer-bound hindered amines. Part I—Bismaleate esters. Polym. Degrad. Stab..

[20] Passaglia E., Coiai S., Cicogna F., Ciardelli F. (2014). Some recent advances in polyolefin functionalization. Polym. Int..

[21] Franssen N.M.G., Reek J.N.H., de Bruin B. (2013). Synthesis of functional ‘polyolefins’: State of the art and remaining challenges. Chem. Soc. Rev..

[22] Cicogna F., Coiai S., Passaglia E., Tucci I., Ricci L., Ciardelli F., Batistini A. (2011). Grafting of Functional Nitroxyl Free Radicals to Polyolefins as a Tool to Postreactor Modification of Polyethylene-Based Materials with Control of Macromolecular Architecture. J. Polym. Sci. Part A Polym. Chem..

[23] Cicogna F., Coiai S., Pinzino C., Ciardelli F., Passaglia E. (2012). Fluorescent polyolefins by free radical post-reactor modification with functional nitroxides. React. Funct. Polym..

[24] Cicogna F., Coiai S., Rizzarelli P., Carroccio S., Gambarotti C., Domenichelli I., Yang C., Dintcheva N.T., Filippone G., Pinzino C. (2014). Functionalization of aliphatic polyesters by nitroxide radical coupling. Polym. Chem..

[25] Cicogna F., Domenichelli I., Coiai S., Bellina F., Lessi M., Spiniello R., Passaglia E. (2016). Azo-aromatic functionalized polyethylene by nitroxide radical coupling (NRC) reaction: Preparation and photo-physical properties. Polymer.

[26] Domenichelli I., Coiai S., Pinzino C., Taddei S., Martinelli E., Cicogna F. (2017). Polymer surface modification by photografting of functional nitroxides. Eur. Polym. J..

[27] Molloy B.M., Johnson K., Ross R.J., Parent J.S. (2016). Functional group tolerance of AOTEMPO-mediated peroxide cure chemistry. Polymer.

[28] Hyslop D.K., Parent J.S. (2013). Dynamics and yields of AOTEMPO-mediated polyolefin cross-linking. Polymer.

[29] Lucarini M., Pedulli G.F. (2010). Free radical intermediates in the inhibition of the autoxidation reaction. Chem. Soc. Rev..

[30] Allen N.S., Kotecha J.L., Parkinson A., Loffelman F.F., Rauhut M.M., Susi P.S. (1985). Photo-stabilising action of a p-hydroxybenzoate light stabiliser in polyolefins: Part III—Antioxidant behaviour and additive/pigment interactions in high density polyethylene. Polym. Degrad. Stab..

[31] Pospíšil J. (1993). Chemical and photochemical behaviour of phenolic antioxidants in polymer stabilization: A state of the art report, part II. Polym. Degrad. Stab..

[32] Gijsman P. (2017). A review on the mechanism of action and applicability of Hindered Amine Stabilizers. Polym. Degrad. Stab..

[33] Denisov E.T., Shestakov A.F. (2015). Reaction between radicals and *N*-alkoxyamines as coordinated cleavage with fragmentation. Russ. J. Phys. Chem. A.

[34] Pilař J., Michálková D., Šedìnková I., Pfleger J., Pospíšil J. (2011). NOR and nitroxide-based HAS in accelerated photooxidation of carbon-chain polymers; Comparison with secondary HAS: An ESRI and ATR FTIR study. Polym. Degrad. Stab..

[35] Marek A., Kaprálková L., Schmidt P., Pfleger J., Humlíček J., Pospíšil J., Pilař J. (2006). Spatial resolution of degradation in stabilized polystyrene and polypropylene plaques exposed to accelerated photodegradation or heat aging. J. Polym. Degrad. Stab..

[36] Kósa C.S., Chmela Š., Pawelke B., Thumer G., Habicher W.D. (2003). New combined hindered phenol/hindered amine stabilizers for polymers based on diphenylmethane-4,4′-diisocyanate. Polym. Degrad. Stab..

[37] Mosnáček J., Chmela Š., Theumerb G., Habicherb W.D., Hrdlovič P. (2003). New combined phenol/hindered amine photo- and thermal-stabilizers based on toluene-2,4-diisocyanate. Polym. Degrad. Stab..

[38] Colwell J.M., Walker J.R., Blinco J.P., Micallef A.S., Graeme G.A., Bottle S.E. (2010). Profluorescent nitroxides: Thermo-oxidation sensors for stabilised polypropylene. Polym. Degrad. Stab..

[39] Calucci L., Cicogna F., Forte C. (2013). Effects of post-reactor functionalization on the phase behaviour of an ethylene-1-octene copolymer studied using solid-state high resolution 13C NMR spectroscopy. Phys. Chem. Chem. Phys..

[40] Mukai K., Yano H., Ishizu K. (1981). ESR studies of a new phenoxyl-nitroxide hetero biradical. Tetrahedron Lett..

[41] Atkinson D., Lehrle R. (1992). A model polymer-bound antioxidant system for lubricant. Eur. Polym. J..

[42] Mendichi R., Giacometti Schieroni A., Pandalai S.G. (2001). Current Trends in Polymer Science.

[43] Murphy J. (2001). Additives for Plastics Handbook.

[44] Mark H.F. (2004). Encyclopaedia of Polymer Science and Technology.

[45] Carroccio S., Rizzarelli P., Zampino D. (2011). A Snapshot of Thermo-Oxidative Degradation Products in Poly(bisphenol A carbonate) by Electrospray Ionization Mass Spectrometry and Matrix-Assisted Laser Desorption Ionization Time of Flight Mass Spectrometry. Macromol. Chem. Phys..

[46] Paine M.R.L., Barker P.J., Blanksby S.J. (2011). Desorption electrospray ionisation mass spectrometry reveals in situ modification of a hindered amine light stabiliser resulting from direct N–OR bond cleavage. Analyst.

[47] Marshall D.L., Christian M.L., Gryn’ova G., Coote M.L., Barkera P.J., Blanksby S.J. (2011). Oxidation of 4-substituted TEMPO derivatives reveals modifications at the 1- and 4-positions. Org. Biomol. Chem..

[48] Thorshaug K., Mendichi R., Boggioni L., Tritto I. (2002). Poly(ethene-*co*-norbornene) Obtained with a Constrained Geometry Catalyst. A Study of Reaction Kinetics and Copolymer Properties. Macromolecules.

[49] Zeynalov E.B., Allen N.S. (2006). Modelling light stabilizers as thermal antioxidants. Polym. Degrad. Stab..

[50] Gardette M., Perthue A., Gardette J.-L., Janecska T., Földes E., Pukánszky B., Therias S. (2013). Photo- and thermal-oxidation of polyethylene: Comparison of mechanisms and influence of unsaturation content. Polym. Degrad. Stab..

[51] Focke W.W., van der Westhuizen I. (2010). Oxidation induction time and oxidation onset temperature of polyethylene in air. J. Therm. Anal. Calorim..

[52] Schmid M., Ritter A., Affolter S. (2006). Determination of oxidation induction time and temperature by DSC. J. Therm. Anal. Calorim..

[53] Pospíšil J., Horák Z., Pilař J., Billingham N.C., Zweifel H., Nešpůrek S. (2003). Influence of testing conditions on the performance and durability of polymer stabilisers in thermal oxidation. Polym. Degrad. Stab..

[54] Pospíšil J., Pilař J., Nešpůrek S. (2007). Exploitation of the complex chemistry of hindered amine stabilizers in effective plastics stabilization. J. Vinyl Addit. Technol..

[55] Luengo C., Allen N.S., Wilkinson A., Edge M., Parellad M.D., Barrio J.A., Ruitz Santa V., Parellada M.D., Barrio J.A. (2006). Synergistic profiles of chain-breaking antioxidants with phosphites and hindered amine light stabilizers in styrene–ethylene–butadiene–styrene (SEBS) block copolymer. J. Vinyl Addit. Technol..

[56] Al-Malaika S., Riasat S., Lewucha C. (2014). Reactive antioxidants for peroxide crosslinked polyethylene. Polym. Degrad. Stab..

[57] Molly B.M., Hyslop D.K., Parent L.S. (2014). Comparative analysis of delayed-onset peroxide crosslinking formulations. Polym. Eng. Sci..

[58] Vieth W.R., Amini M.A., Hopfenberg H. (1974). Permeability of Plastic Films and Coatings.

