# Supplementary material for: Grafting of Hindered Phenol Groups onto Ethylene/α-Olefin Copolymer by Nitroxide Radical Coupling
Source: Polymers (Basel). 2017 Dec 4;9(12):670. doi: 10.3390/polym9120670 (PMC6418857; doi:10.3390/polym9120670)
Supplement: Supplementary file 1 [file polymers-09-00670-s001.pdf]

Supplementary Materials

Grafting of Hindered Phenol Groups onto Ethylene/α-Olefin Copolymer by Nitroxide Radical Coupling

Serena Coiai [1], Francesca Cicogna [1,*], Chengcheng Yang [1], Veronika Tempesti [1], Sabrina Carola Carroccio [2,3], Giuliana Gorrasi [4], Raniero Mendichi [5], Nadka Tz. Dintcheva [6] and Elisa Passaglia [1]

[1] Istituto di Chimica dei Composti Organo Metallici (ICCOM), Consiglio Nazionale delle Ricerche, SS Pisa, Via G. Moruzzi 1, 56124 Pisa, Italy; serea.coiai@pi.iccom.cnr.it (S.C.); francesca.cicogna@pi.iccom.cnr.it (F.C.); chengchengyoung@gmail.com (C.Y.); v.tempesti@outlook.it (V.T.); passaglia@pi.iccom.cnr.it (E.P.)

[2] Istituto per i Polimeri, Compositi e Biomateriali (IPCB), Consiglio Nazionale delle Ricerche, SS Catania, Via P. Gaifami 18, 95126 Catania, Italy; sabrinacarola.carroccio@cnr.it

[3] Istituto per la microelettronica e microsistemi (IMM), Consiglio Nazionale delle Ricerche, SS Catania (Università), Via S. Sofia 64, 95123 Catania, Italy

[4] Dipartimento di Ingegneria Industriale, Università degli studi di Salerno, Via Giovanni Paolo II, 132, 84084 Fisciano (SA), Italy; ggorrasi@unisa.it

[5] Istituto per lo studio delle macromolecule (ISMAC), Consiglio Nazionale delle Ricerche,, Via A. Corti 12, 20133 Milano, Italy; mendichi@ismac.cnr.it

[6] Dipartimento di Ingegneria Civile, Ambientale, Aerospaziale, dei Materiali, Università di Palermo, Viale delle Scienze, Ed. 6, 90128 Palermo, Italy; nadka.dintcheva@unipa.it

* Correspondence: francesca.cicogna@pi.iccom.cnr.it; Tel.: +39-050-3152-3393

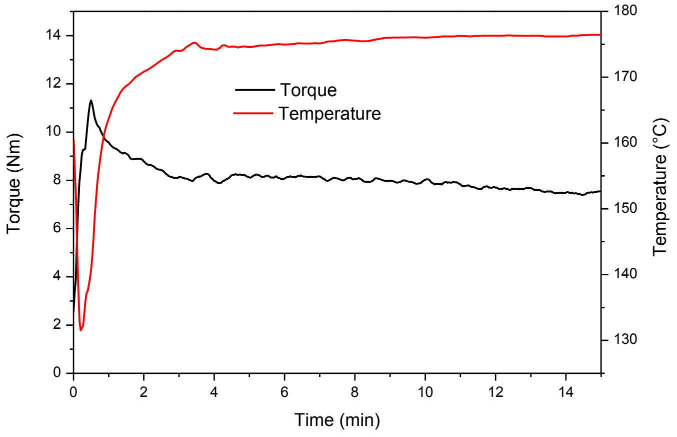

Figure S1. Torque curve and temperature profile recorded during the functionalization of EOC with BHB-T.

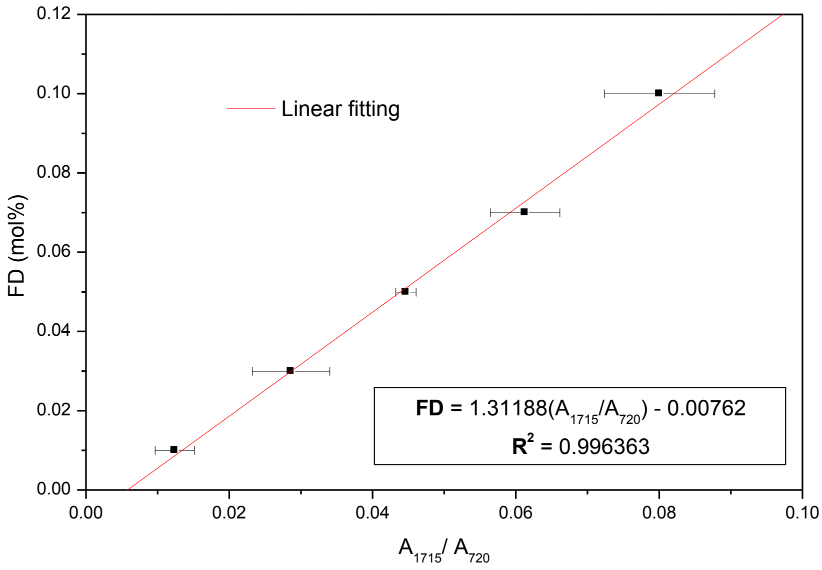

Figure S2. Calibration curve for the determination of the FD of EOC-g-(BHB-T) samples.

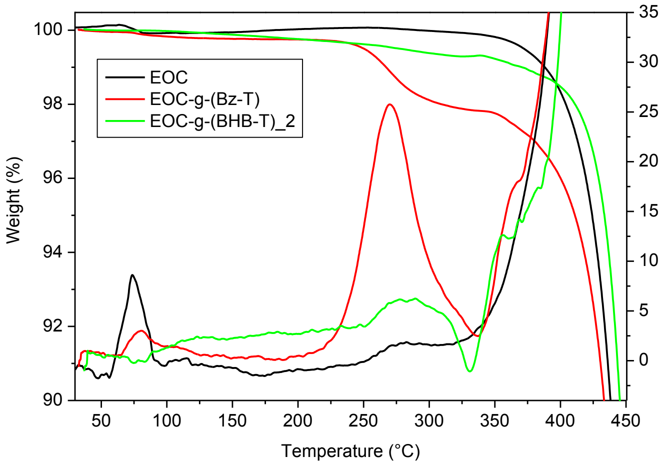

Figure S3. TGA thermograms and their first derivative of EOC-g-(Bz-T) and EOC-g-(BHB-T)_2.

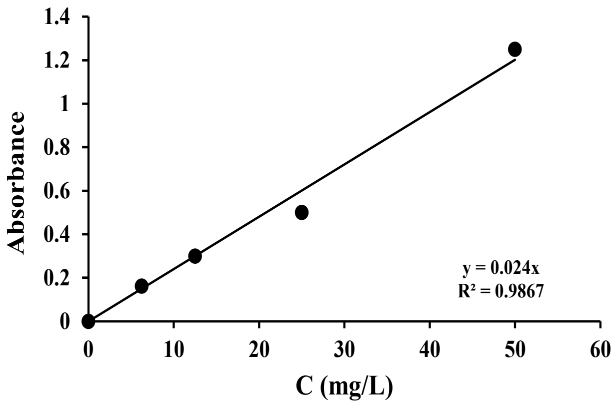

Figure S4. Calibration curve for the determination of the amount of CY and BHB-T released from EOC/CY2 and EOC-g-(BHB-T)_2 during migration tests.

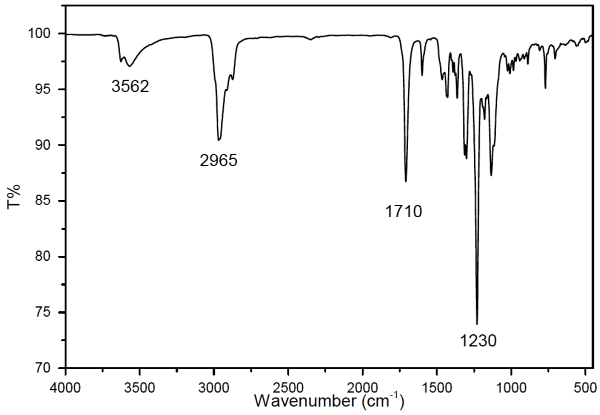

Figure S5. FT-IR spectrum of 3,5-di-tert-butyl-4-hydroxybenzoyl-2,2,6,6-tetramethylpiperidine-1-oxyl radical (BHB-T)

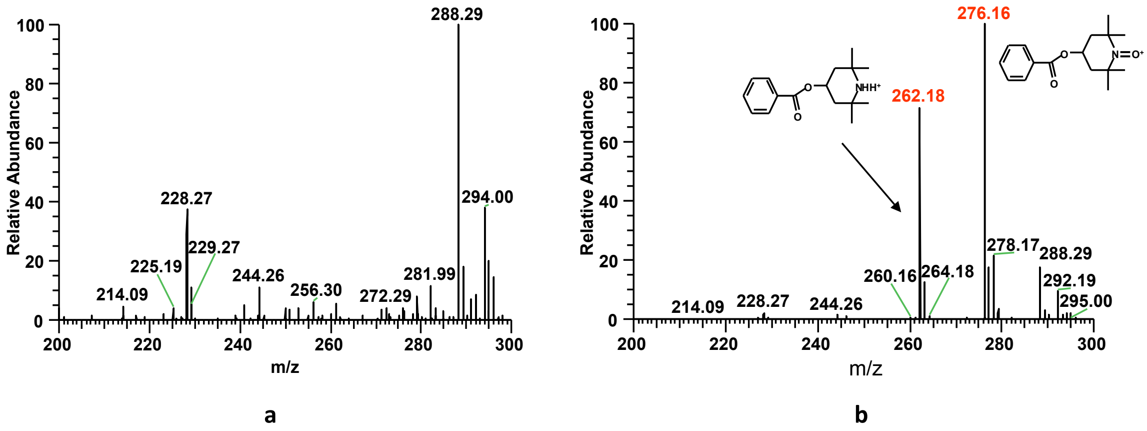

Figure S6. ESI mass spectra registered in positive mode, in the mass range 200-300 m/z, of the products extracted from EOC-g-(Bz-T) virgin sample (a) and photo-oxidized for 6 days (b).

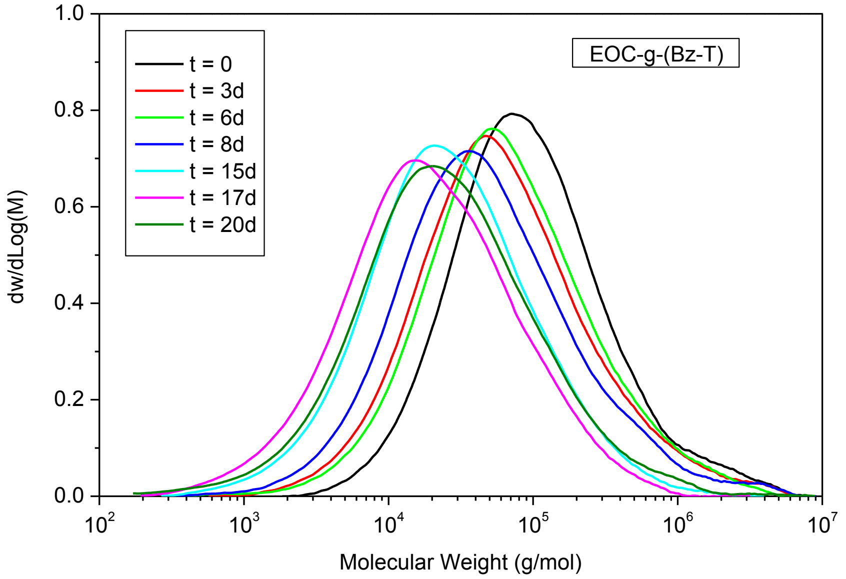

Figure S7. Differential MWD of EOC-g-(Bz-T) before and after different UV irradiation times.

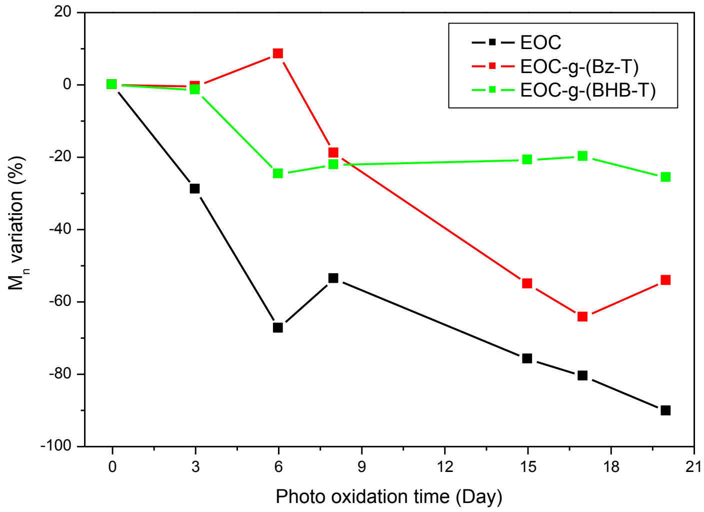

Figure S8. Mn variation as a function of irradiation time.

Table S1. SEC-DV characterization of EOC, EOC-g-(Bz-T) and EOC-g-(BHB-T)_2 during photo-oxidation

Sample	Irradiation day	M_w kg/mol	M_n kg/mol	M_w/M_n	[η] dL/g	Mw Var. %	[η] Var. %
EOC_0	0	179.7	86.9	2.1	0.91	0	0
EOC_3	3	167.2	61.8	2.7	0.79	-6.9	-13.2
EOC_6	6	144.4	28.4	5.1	0.56	-19.5	-38.5
EOC_8	8	162.6	40.3	4.0	0.64	-9.6	-29.7
EOC_15	15	118.9	21.0	5.7	0.47	-33.8	-48.3
EOC_17	17	131.6	16.9	7.8	0.47	-26.7	-48.3
EOC_20	20	62.0	8.5	7.3	0.30	-65.5	-67.0
EOC-g-(Bz-T)_0[1]	0	246.0	43.3	5.7	0.81	0	0
EOC-g-(Bz-T)_3	3	192.8	43.2	4.5	0.74	-21.6	-8.6
EOC-g-(Bz-T)_6	6	182.0	47.1	3.9	0.70	-26.0	-13.6
EOC-g-(Bz-T)_8	8	181.6	35.2	5.2	0.70	-26.2	-13.6
EOC-g-(Bz-T)_15	15	82.6	19.5	4.2	0.42	-66.4	-48.1
EOC-g-(Bz-T)_17	17	65.3	15.5	4.2	0.43	-73.4	-46.9
EOC-g-(Bz-T)_20	20	92.8	19.9	4.7	0.57	-62.3	-29.6
EOC-g-(BHB-T)_2_0	0	175.9	82.7	2.1	0.89	0	0
EOC-g-(BHB-T)_2_3	3	199.6	81.5	2.4	0.89	+14.0	0
EOC-g-(BHB-T)_2_6	6	231.0	62.3	3.7	0.96	+32.0	+7.9
EOC-g-(BHB-T)_2_8	8	249.6	64.4	3.9	0.93	+42.6	+4.5
EOC-g-(BHB-T)_2_15	15	310.1	65.5	4.7	0.95	+77.2	+6.7
EOC-g-(BHB-T)_2_17	17	293.5	66.3	4.4	0.96	+67.7	+7.9
EOC-g-(BHB-T)_2_20	20	294.7	61.5	4.8	0.92	+68.4	+3.4

[1] EOC-g-(Bz-T) samples were analyzed by a different SEC column set (See Experimental)

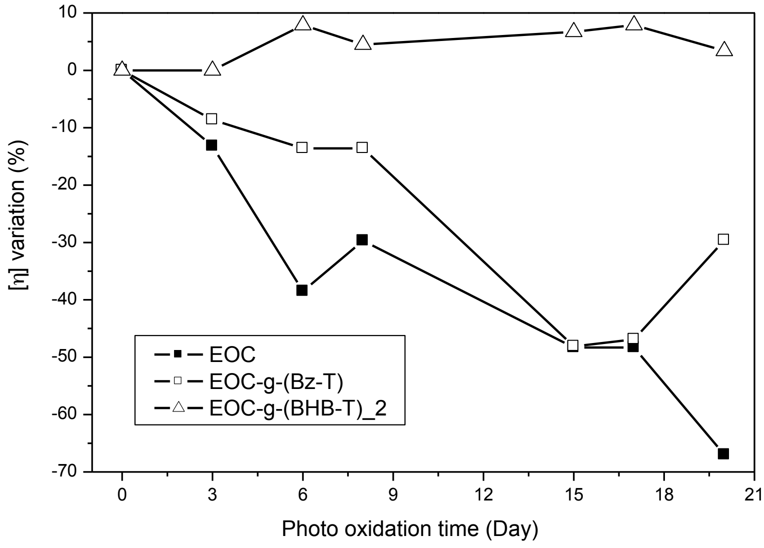

Figure S9. Intrinsic viscosity variation as a function of irradiation time.

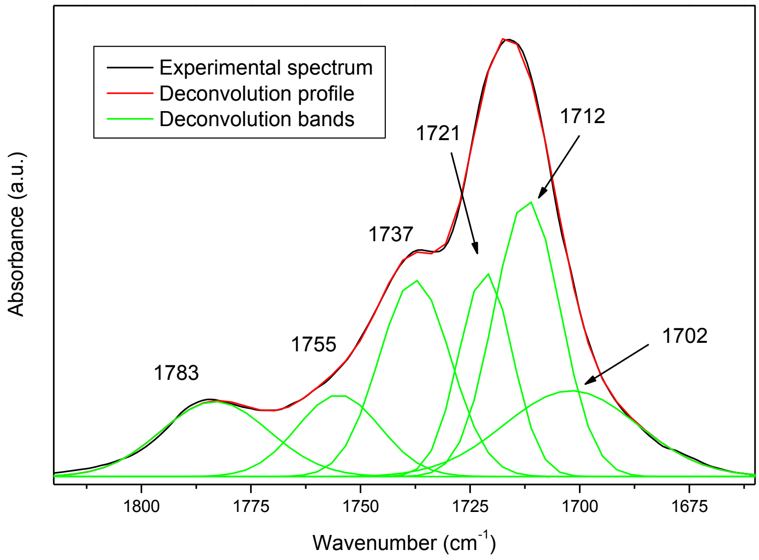

Figure S10. Deconvoluted IR spectrum of EOC thermo-oxidized for 30 days in the absorption region of carbonyl group.

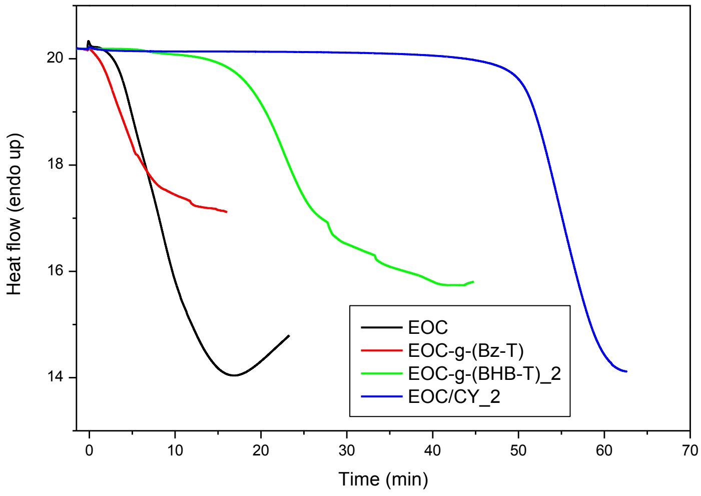

Figure S11. Oxidation induction time (OIT) curves of pristine EOC, EOC functionalized with Bz-T and BHB-T and EOC mixed with CY. Acquisition temperature 190°C, oxygen flow 50 ml/min.

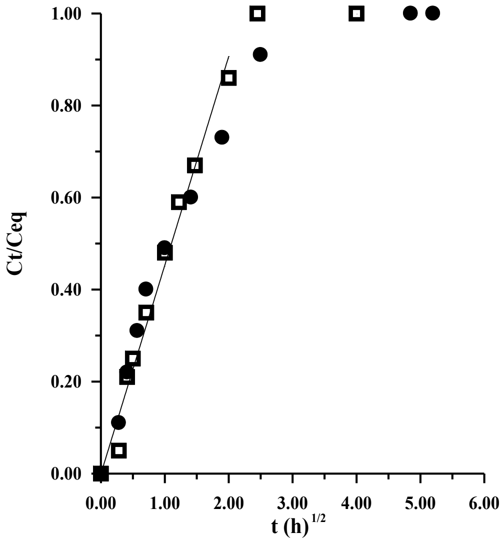

Figure S12. Ct/Ceq vs. square root of time (h) of EOC in ethanol for samples: (•) EOC-g-(BHB-T)_2; () EOC/CY_2.
